# Symptomatic Calcified Uterine Fibroids Refractory to Repeat Uterine Artery Embolization: A Case Report

**DOI:** 10.7759/cureus.61081

**Published:** 2024-05-25

**Authors:** Christopher Baker, Pooja Indir, Kendall Handy, Jefferson Jones

**Affiliations:** 1 Osteopathic Medicine, Philadelphia College of Osteopathic Medicine, Moultrie, USA; 2 Family Medicine, Piedmont Columbus Midtown, Columbus, USA; 3 Obstetrics and Gynecology, Piedmont Columbus Midtown, Columbus, USA

**Keywords:** pelvic pain, myomectomy, uterine artery embolization, abnormal uterine bleeding, uterine fibroids

## Abstract

Uterine leiomyomas, also known as uterine fibroids, are a commonly encountered condition with a diverse clinical presentation. Uterine fibroids are benign, smooth muscle tumors of the uterus arising from a single myometrial cell. The presentation can vary from asymptomatic incidental findings to causing a wide array of gynecological symptoms, including abnormal uterine bleeding, infertility, chronic pelvic pain, and bulk-related symptoms. There are several management approaches depending on the patient's clinical manifestations and goals.

This is a unique case of a patient with symptomatic calcified uterine fibroids refractory to medical management and two uterine artery embolizations presenting with persistent abnormal uterine bleeding and chronic pelvic pain. Preservation of the uterus was desired, so an open myomectomy was subsequently performed. The patient was asymptomatic at two weeks follow-up, and further follow-up was unable to be obtained.

When considering interventions for symptomatic uterine fibroids, it is essential to consider the patient's preference for uterine-sparing methods and desire to preserve fertility. It is necessary that all modes of treatment and their potential future implications be discussed so that patients can make well-informed decisions regarding all aspects of their care. Further studies are needed comparing the outcomes of uterine-sparing interventions for symptomatic uterine fibroids so that the best possible shared decision-making can take place.

## Introduction

This case illustrates the presentation and course of management of a patient with large symptomatic uterine fibroids refractory to medical therapy and two uterine artery embolizations (UAEs). It has been estimated that up to 70% of women will develop uterine fibroids by menopause [1]. The prevalence, however, is likely underestimated due to them commonly being asymptomatic and remaining undiagnosed [1]. Significant risk factors for developing uterine fibroids include age, race, family history of uterine fibroids, and hypertension [1-10]. The risk of uterine fibroids is up to 10 times greater in women in their 40s and 50s compared to women in their 20s [8], greater than three times the risk in women with a family history of uterine fibroids [9], and almost five times the risk in patients with hypertension [10]. Uterine fibroids are up to 2-3 times more prevalent in black women as compared to white women [4-6]. However, factors other than race itself are thought to contribute to this difference such as environmental exposures, lifestyle, perceived racism, psychosocial stress, and diet [11,12]. Protective factors against uterine fibroids include menopause, multiparity (>3), and history of oral contraceptive use [1-3,13,14]. The clinical presentation of uterine fibroids is related to the location, size, and number of tumors, which has been further classified using the International Federation of Gynecology and Obstetrics (FIGO) system [15]. Uterine fibroid location can be described as submucosal, intramural, subserous, or a combination of the aforementioned with additional descriptors such as intramural thickness and pedunculated tumors [15]. These classifications, along with presentation and patient preference, can help guide treatment options. Common symptoms of uterine fibroids include abnormal uterine bleeding, commonly experienced as heavy or prolonged menstrual bleeding; bulk-related symptoms such as pelvic pressure, pelvic pain, urinary tract obstruction, bowel obstruction, or venous compression; and reproductive dysfunction, including infertility and miscarriages [3,16]. However, many patients will remain asymptomatic, and only an estimated 25% of uterine fibroids will be clinically significant enough to require intervention [1-3,16]. Among symptomatic patients, heavy or prolonged bleeding and menstrual cramps are the most common symptoms experienced by 26-29% of patients [17,18]. Management options vary depending on the type and severity of symptoms experienced. For patients with mild symptoms, expectant management is indicated. For patients with bleeding symptoms, medical management can include gonadotropin-releasing hormone (GnRH) antagonists, GnRH agonists, tranexamic acid, contraceptive steroids, and levonorgestrel-releasing intrauterine devices. In patients experiencing bulk symptoms or failed medical management, procedural interventions can include UAE, radiofrequency ablation, focused ultrasound (US) surgery, endometrial ablation, myomectomy, and hysterectomy. It is pertinent that shared decision-making be utilized when deciding treatment options, specifically considering the patient's desire for future pregnancies or preference for uterine-preserving methods, given comparative effectiveness data are lacking for leiomyoma management options [3]. UAE involves delivering an embolic agent (polyvinyl alcohol or microspheres produced from an acrylic polymer and impregnated with porcine gelatin) to both uterine arteries, leading to the devascularization and subsequent involution of uterine fibroids. UAEs are effective in reducing symptom severity and improving patient satisfaction; however, the rates of reintervention have been reported as 14.4% and even up to 20% at five years [19,20]. Myomectomies can either be performed as hysteroscopic, laparoscopic, or open myomectomies. The procedure preserves the uterus but removes or debulks the uterine fibroids and may involve morcellation of the specimen. The risk of reintervention following a myomectomy for uterine fibroids has been reported as 23% at five years and up to 30% at seven years [3,21]. When comparing surgery versus embolization, more adverse events occurred in the surgical group during the hospital stay, while the opposite was true after discharge [22]. The risk of reintervention within 32 months of a UAE has been reported to be 2-10 times greater than that following a myomectomy or hysterectomy [3,22-25].

## Case presentation

A 42-year-old African American female presented for persistent abnormal uterine bleeding (AUB) and the onset of chronic pelvic pain following a UAE one year ago for symptomatic uterine leiomyomas. This was her second UAE, with the first UAE being performed 12 years prior. Thelarche onset was at 11 years of age, while menarche onset was at 12 years of age. Before the onset of her AUB at 29 years of age, she experienced regular menstrual cycles with 3-4 days of bleeding, requiring 6-7 standard tampons per day at peak flow. She is G3P0030 with three spontaneous first-trimester abortions occurring at 14, 18, and 20 years of age. She reports a remote history of pelvic inflammatory disease at 15 years old due to *Chlamydia trachomatis* that was treated with no further complications. She is up to date on cervical cancer screening with her last two Pap smears resulting negative for cervical intraepithelial neoplasia (CIN) or malignancy and negative for human papillomavirus (HPV). She has a past medical history of anxiety, major depressive disorder, and uterine fibroids. Her only regular medication is fluoxetine, with trials of lurasidone and quetiapine in the past. No other significant medical conditions were reported. She has never required a blood transfusion for anemia. She reports no other surgical history. She denies any history of tobacco use, alcohol use, or any use of recreational or illicit substances. She has a family history of hypertension in her father and intracranial neoplasm in her maternal aunt. She is unaware of any family history of uterine fibroids, breast cancer, ovarian cancer, or endometrial cancer. She first noticed the onset of symptoms at 29 years of age, described as a fullness in her lower abdomen and pelvis as well as prolonged menstrual bleeding that progressed from three days of bleeding to seven days of bleeding at the start of each cycle. She does not recall experiencing any overt pelvic pain, urinary symptoms, or bowel symptoms. She sought out care and was diagnosed with uterine fibroids. She underwent her first UAE at the age of 30 for large symptomatic uterine fibroids causing prolonged menstrual bleeding. She did not undergo any other therapy for her symptoms at the time, including medical management of any sort. Following her first UAE, she noticed a decrease in the pelvic fullness she had been experiencing but no changes to the prolonged menstrual bleeding for seven days at the start of each cycle. In addition, she noted the onset of a continuous clear non-odorous vaginal discharge. Over the next couple of years, the constant clear vaginal discharge persisted with the onset of light intermenstrual vaginal bleeding described as spotting. Her AUB progressively worsened with heavier bleeding at the start of each cycle and continuous intermenstrual bleeding. At its peak, when she was 40 years of age, she described the AUB as prolonged heavy bleeding at the start of her menstrual cycle for seven days requiring at least five ultra-absorbent tampons per day with unremitting light intermenstrual bleeding until the beginning of her next cycle. Her cycles continued to occur regularly every 28-30 days. At this time, she again sought care for her symptoms. Over the next year, she was prescribed Junel (norethindrone/ethinyl estradiol) and Myfembree (relugolix/estradiol/norethindrone acetate) that she took to minimally relieve her AUB. She had a pelvic magnetic resonance imaging (MRI) showing large uterine fibroids and the uterus measuring 14.5×8.4×7.4 cm.

**Figure 1:**
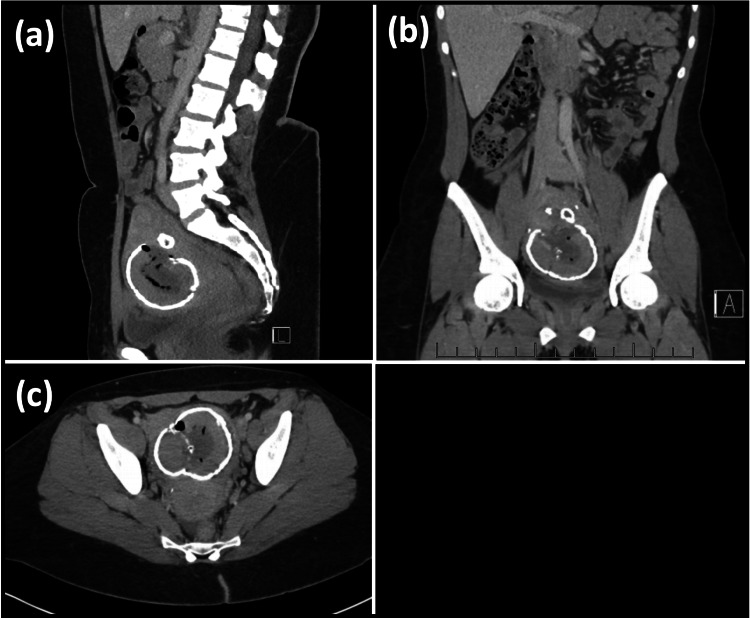
CT of the abdomen/pelvis with contrast showing calcified fibroid measuring 6.8×7.2×6.9 cm: (a) sagittal view, (b) coronal view, and (c) axial view CT: computed tomography

**Figure 2:**
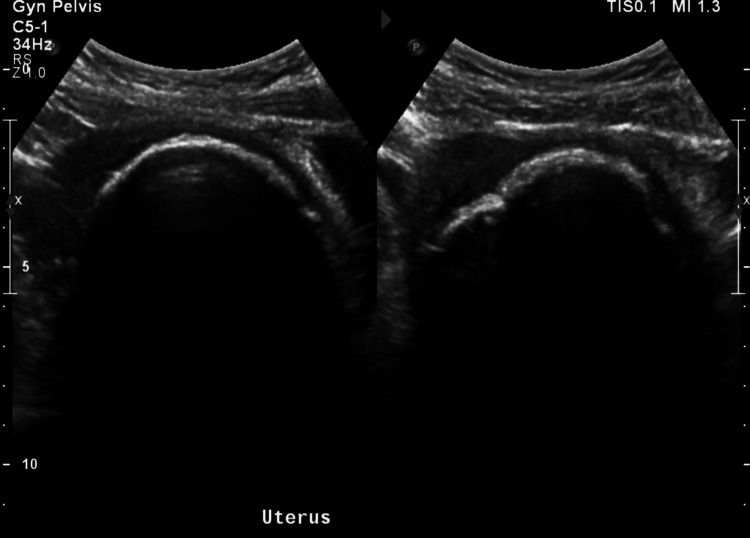
Pelvic US: sagittal view (left) and transverse view (right) US: ultrasound

Surgical management was discussed with the patient who desired preservation of the uterus. She underwent her second UAE at the age of 41 for large symptomatic uterine fibroids causing prolonged menstrual bleeding and intermenstrual bleeding. Following the procedure, her AUB did not improve, and she continued to experience prolonged heavy menstrual bleeding with intermenstrual bleeding and the onset of chronic pelvic pain. Within two months of the UAE, she had three emergency department visits for lower abdominal pain, dysuria, and non-odorous yellow vaginal discharge. Imaging studies were performed in the emergency department, including a computed tomography (CT) of the abdomen/pelvis with contrast (Figure 1) and a pelvic US (Figure 2).

**Figure 3:**
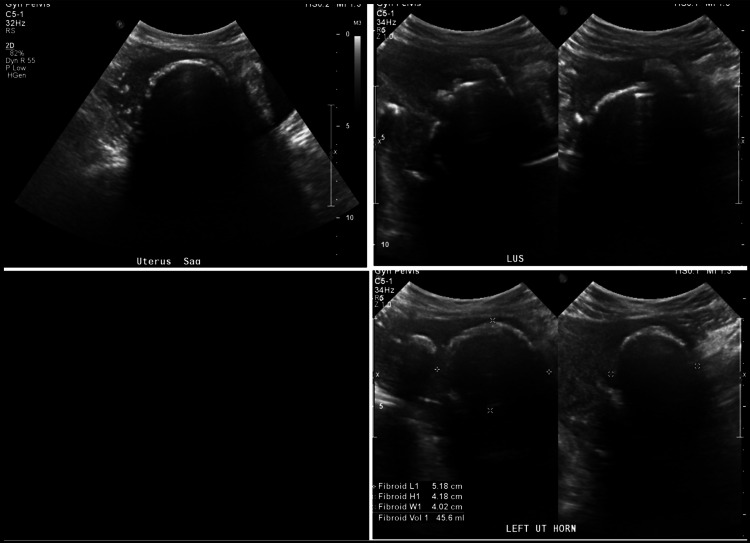
Multiple views of the pelvic US showing calcified fibroids measuring 5.2×4.2×4.0 cm and 5.1×5.1×5.9 cm. Does not appear significantly changed from prior CT of the abdomen/pelvis with contrast about one year prior. Similar in size to the pelvic US from about one year ago US: ultrasound

She was covered with empiric antibiotics for potential endometritis and treated multiple times for an uncomplicated urinary tract infection. She had a postoperative pelvic MRI performed two months following the UAE. The pelvic MRI showed the uterus was overall mildly decreased in size compared to the pelvic MRI 2.5 months prior. The largest fibroid continued not to demonstrate post-contrast enhancement similar to the prior pelvic MRI. The non-odorous vaginal discharge resolved within three months, but her pelvic pain persisted, and the AUB remained unchanged. She was prescribed tranexamic acid, which provided only mild relief of her AUB; further surgical interventions were discussed at that time. She still desired preservation of the uterus, and through shared decision-making, she agreed to an open myomectomy. It was discussed thoroughly that the myomectomy would not be performed to preserve potential fertility, especially in the setting of two past UAEs, but that it would be performed for the management of symptoms. A pelvic US was performed prior to the myomectomy (Figure 3) that showed multiple calcified uterine fibroids that did not appear significantly changed from prior CT of the abdomen/pelvis with contrast and the pelvic US from one year prior.

**Figure 4:**
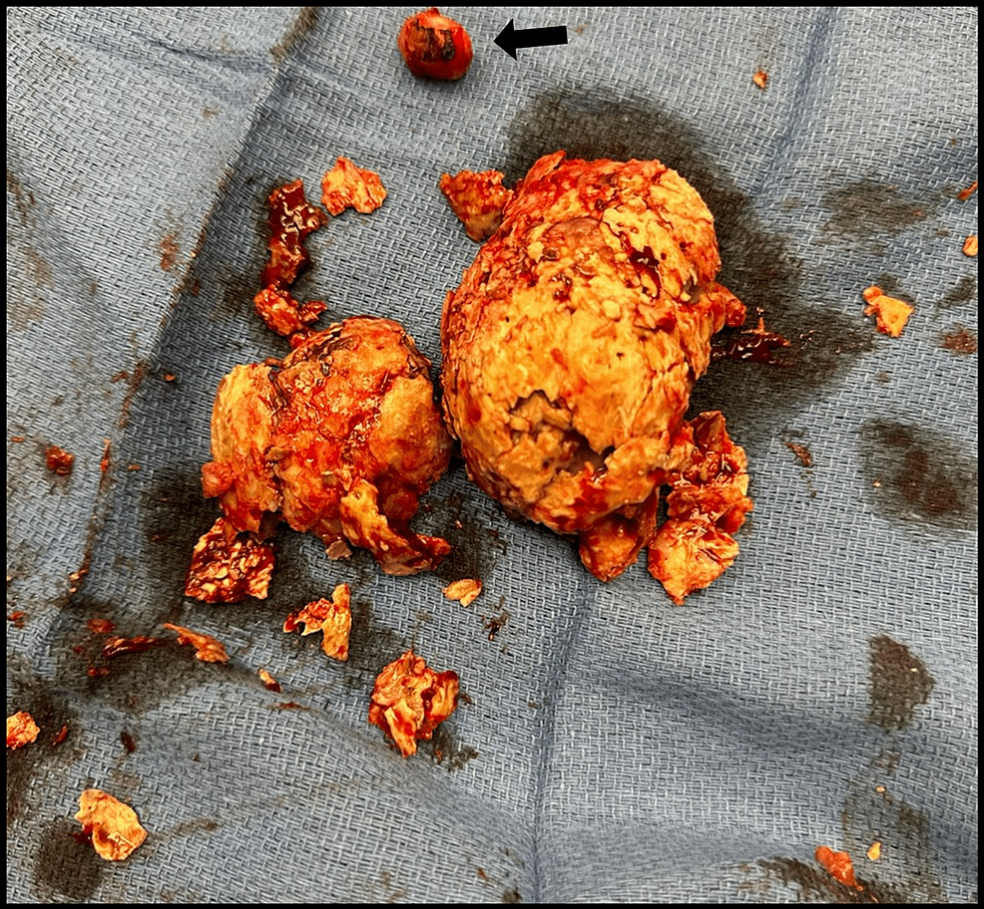
Gross intraoperative photograph of the submucosal calcified fibroid and pedunculated subserosal fibroid (black arrow) removed during open myomectomy

She underwent an open myomectomy at the age of 42 for large symptomatic uterine fibroids causing prolonged menstrual bleeding and intermenstrual bleeding refractory to medical management and two prior UAEs. The open myomectomy was performed without complications. At the time of surgery, the patient had a myomatous uterus approximately 8-9 weeks in size. A 2 cm pedunculated subserosal fibroid was removed from the fundus of the uterus. Ten units of pitressin solution in 100 cc of normal saline was injected into the firm 5-6 cm submucosal fibroid. Electrocautery was then used to dissect down to the fibroid, which was removed without difficulty. The remaining portions of calcified tissue were then removed. The endometrial cavity was entered secondary to the fibroid's submucosal location. The endometrium was repaired, followed by the serosal surface. The surgical specimen removed (Figure 4) was later weighed and measured at 87 g and 8.5×8.0×3.3 cm. It was calcified and firm with a disrupted irregular yellow-tan to purple-red appearance.

**Figure 5:**
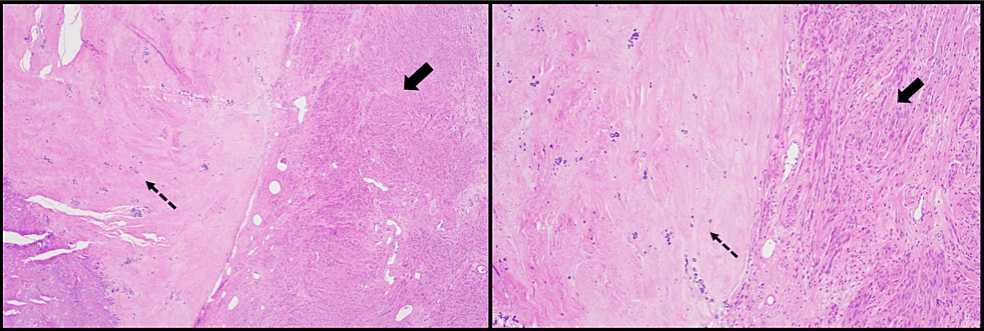
The slide on the left (H&E, 4×) shows features of a spindle cell lesion most consistent with a leiomyoma. The solid black arrow points to the viable spindle cells. The dotted black arrow indicates calcifications and hyaline degeneration. The slide on the right (H&E, 10×) is similar to the last one but at a higher power

**Figure 6:**
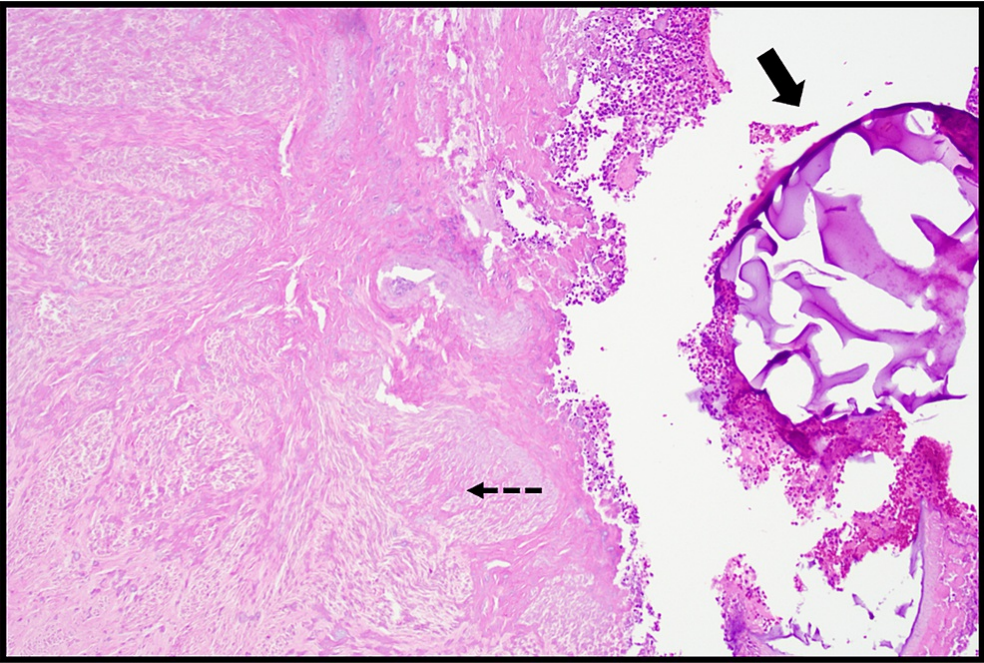
The dotted black arrow shows an area of infarct-type necrosis with adjacent acute inflammation. The solid black arrow points to the basophilic foreign material, likely embolization material (H&E, 10×)

The surgical specimen was sent to pathology, where it was confirmed to be a uterine leiomyoma, negative for cytologic atypia and malignancy. Microscopic examination (Figure 5 and Figure 6) showed infarct-type necrosis, calcifications, and mild chronic and acute inflammation. Basophilic foreign material was observed within vessels, consistent with embolization material.

The patient had no major post-op complications while in the hospital and was discharged the following afternoon. She did experience mild vaginal bleeding the evening of the operation, as expected, but this resolved by the next morning. At two weeks follow-up, the patient's surgical incision was healing well with no erythema, tenderness, or dehiscence. The patient had no complaints at that time. She denied vaginal bleeding, vaginal discharge, abdominal pain, fever, dysuria, or other symptoms.

## Discussion

Uterine fibroids are very common in reproductive-age females. For some, they are entirely asymptomatic, and others require treatment [26]. UAE was introduced for the treatment of symptomatic uterine fibroids in 1995 [27]. UAE provides a minimally invasive and uterine-sparing treatment option. Ideal candidates for UAE include patients with heavy menstrual bleeding or dysmenorrhea caused by intramural fibroids, who are premenopausal, and who have no desire for future pregnancy [28]. The American College of Obstetricians and Gynecologists states that the effect of UAE on pregnancy remains understudied but makes no recommendation of whether desire for a future pregnancy is a contraindication [3]. There is concern that poor uterine perfusion following UAE would negatively impact fertility and result in obstetric complications or adverse fetal effects. It has been shown that most patients (73-90%) reported improvement or disappearance of heavy menstrual bleeding symptoms up to 10 years after treatment with UAE. Subsequent hysterectomy for failure or recurrence of symptoms after UAE was reported to be 27% (51 of 187 patients) at five years in a meta-analysis of four randomized trials [29]. UAE may decrease ovarian reserve and even result in premature ovarian failure. After UAE to treat fibroids, there's a chance the fibroids will recur. Studies show that 32% of people had symptoms associated with uterine fibroids after five years [30]. A second round of UAE may be needed to treat your symptoms if your fibroids return. Typically, a repeat UAE is indicated in patients who experience recurrent fibroids, usually in the setting of regrowth of incompletely infarcted tumors. A small study showed 90% of women had symptom control following a repeat UAE [31]. In this study, between the initial and subsequent UAE, half the cases showed increases in uterine and tumor volume, but half showed volume decreases. This finding supports that symptoms can recur even when tumor size decreases following the primary UAE. It is suspected that the recurrence of symptoms is determined by the extent of perfusion of the fibroids, regardless of size [31].

Traditionally, myomectomy has been the procedure of choice for patients with fibroids who desire future pregnancy or preservation of the uterus [32]. Younger patients are at a greater risk of fibroid recurrence following myomectomies; subsequent surgeries, including repeat myomectomies, may be necessary [21]. This potential outcome should be discussed with the patient and considered when deciding whether to pursue an initial myomectomy versus a hysterectomy.

## Conclusions

The patient in this case report had symptomatic uterine fibroids refractory to multiple treatments, including medical management and two UAEs. An open myomectomy was subsequently performed. We were unable to obtain long-term follow-up. When considering interventions for symptomatic uterine fibroids, it is essential to consider the patient's preference for uterine-sparing methods and desire to preserve fertility. Discussions need to occur with the patient describing the surgical options, including UAE, myomectomy, and hysterectomy. The potential outcomes of such interventions need to be discussed in depth and the lack of quality evidence available on the effects of a UAE on future fertility. More studies are needed comparing the outcomes of interventions on fertility and complications.
